# The Ultrasound Renal Stress Test for the Assessment of Functional Renal Reserve in Kidney Transplantation: A Pilot Study in Living Donors

**DOI:** 10.3390/jcm13020525

**Published:** 2024-01-17

**Authors:** Federico Nalesso, Francesca K. Martino, Marco Bogo, Elisabetta Bettin, Marianna Alessi, Lucia F. Stefanelli, Cristina Silvestre, Lucrezia Furian, Lorenzo A. Calò

**Affiliations:** 1 Department of Medicine, Nephrology-Dialysis-Kidney Transplant Unit, University of Padua, 35128 Padua, Italy; francesca.martino.k@gmail.com (F.K.M.); marco.bogo@studenti.unipd.it (M.B.); luciafederica.stefanelli@aopd.veneto.it (L.F.S.); renzcalo@unipd.it (L.A.C.); 2 Department of Surgical Oncological and Gastroenterological Sciences, Kidney and Pancreas Transplant Unit, University of Padua, 35128 Padua, Italy; cristina.silvestre@aopd.veneto.it (C.S.); lucrezia.furian@unipd.it (L.F.)

**Keywords:** renal functional reserve (RFR), living kidney donation, Intra-Parenchymal Renal, Resistive Index Variation (IRRIV), Renal Stress Test (RST), nephrectomy, kidney transplants, AKI, CKD

## Abstract

In the evolving landscape of nephrology and kidney transplants, assessing renal functional reserve (RFR) in living kidney donors is essential for ensuring donor safety and successful transplantation. This study explores the use of the Intra-Parenchymal Renal Resistive Index Variation (IRRIV) test, a novel non-invasive method, to measure RFR in living donors. Our observational study included 11 participants undergoing living kidney donations, evaluated using the IRRIV-based Renal Stress Test (RST) before and 12 months post-nephrectomy. The study demonstrated significant changes in creatinine and eGFR CKD-EPI levels post-donation, with an average creatinine rise from 69 to 97 µmol/L and a reduction in eGFR from 104 to 66 mL/min/1.73 m^2^. These variations align with the expected halving of nephron mass post-nephrectomy and the consequent recruitment of RFR and hyperfiltration in the remaining nephrons. This pilot study suggests that the IRRIV-based RST is a practical, safe, and reproducible tool, potentially revolutionizing the assessment of RFR in living kidney donors, with implications for broader clinical practice in donor eligibility evaluation, even in borderline renal cases. Furthermore, it confirms the feasibility of RST in living kidney donors and allows us to assess the sample size in 48 donors for a future study.

## 1. Introduction

The importance of assessing renal functional reserve (RFR) in living donors for kidney transplantation is crucial, both for the safety of the donor and the success of the transplant [1]. RFR refers to the capacity of the kidneys to increase their function in response to physiological or pathological stress [2,3].

In the context of living kidney donation, evaluating RFR can provide valuable insights [4]. The primary concern in living kidney donation is the long-term health and safety of the donor. By assessing RFR, clinicians can estimate how well the remaining kidney will compensate after donation. A robust RFR suggests a lower risk of future renal complications in the donor and a low risk of developing Chronic Kidney Disease (CKD). The RFR of the donor may also correlate with the future performance of the transplanted kidney. A healthy kidney with a high RFR is more likely to function effectively in the recipient with less complications related to the transplant condition. Measuring the RFR can help in strategizing against the risk for potential donors, especially those who might be at the borderline of acceptance due to age, mild hypertension, or other factors that do not outright disqualify them, but raise concerns. Long-term studies indicate that living kidney donors generally maintain adequate kidney function [5,6] demonstrating the safety of living donor kidney transplants. However, understanding the RFR can help in more accurately predicting and monitoring outcomes to implement and tailor donors’ follow-ups to prevent complications and improve outcomes [7,8].

Methods for assessing RFR may include stress tests like protein loading [9,10], the IRRIV test [11], or pharmacological stimuli [3], and measuring changes in GFR or other markers of kidney function. However, it is important to note that while the concept of RFR is valuable, its practical application and standardized measurement in the context of living kidney donation are still areas of ongoing research. Samoni et al. [11] investigated the use of the IRRIV (Intraparenchymal Renal Resistive Index Variation) as a non-invasive method to measure RFR. Classically, the RFR represented an increase in the glomerular filtration rate after a protein load, which was measured using a protein loading test, while the IRRIV was assessed by the application of mechanical pressure on the abdomen of healthy volunteers to compress renal vessels resulting in reduced blood flow and the activation of an autoregulatory mechanism, measurable by a decrease in the renal resistive index (RRI) using ultrasound. The findings demonstrated a significant correlation between the IRRIV and RFR, suggesting that the IRRIV could be used as a “stress test” for the rapid assessment of RFR and to establish any renal susceptibility to various exposures and the consequent risk of acute kidney injury (AKI). Regarding these results, thirty healthy adult volunteers were enrolled, evenly split between males and females, with an average age of 38 years. The measured RFR values ranged from −1.9 to 59.7 mL/min/1.73 m^2^, with an average value of 28.9 mL/min/1.73 m^2^. The average baseline RRI was 0.61, which decreased to 0.49 during abdominal pressure. The average IRRIV was 19.6%, ranging from 3.1% to 29.2%. A significant correlation was observed between the RFR and IRRIV (74.16%, *p* < 0.001) suggesting that the IRRIV could be a reliable indicator for RFR.

Samoni et al. [12] discussed that serum creatinine (sCr) values are not accurate markers of renal function when GFR is above 60 mL/min/1.73 m^2^, as they remain normal until up to 50% of nephrons are lost. To evaluate this condition, the RFR is typically assessed using protein load tests. However, these tests can be cumbersome, especially in situations requiring quick diagnosis or screening of large populations. The study proposes the use of IRRIV [11] as an alternative method. The results of the study involved 47 healthy subjects with Caucasian heritage. The mean baseline sCr was 0.85 ± 0.19 mg/dL, corresponding to an estimated baseline GFR of 98.6 ± 23.7 mL/min/1.73 m^2^. The renal functional reserve for the group was calculated to be 29.7 ± 19.1 mL/min/1.73 m^2^. The study found a significant Pearson correlation coefficient of 0.83 between the RFR and IRRIV. The concordance between the RFR and IRRIV was observed in 95.7% of subjects, with a negative IRRIV test accurately predicting the absence of a RFR in five subjects, and a positive IRRIV test correctly predicting the presence of a RFR in 40 subjects. The sensitivity, specificity, positive predictive value, and negative predictive value of the IRRIV test were remarkably high. The study concluded that the IRRIV test is a reliable predictor of renal functional reserve, indicated by a ROC-AUC score of 0.86. In the healthy population, the IRRIV proves to be a valid, effective, and reproducible tool for the study of RFR by identifying possible situations of increased risk of AKI development.

Samoni et al. [13] investigated the relationship between the IRRIV and RFR in patients undergoing elective cardiac surgery, and their utility in predicting AKI and subclinical AKI post-surgery. Thirty-one patients scheduled for cardiac surgery underwent both the protein loading test (to measure RFR) and the IRRIV test two days before surgery. Pearson correlation analysis tested the correlation between the IRRIV and RFR, and logistic regression analysis evaluated the association between these measures and the incidences of AKI and subclinical AKI post-surgery. A strong correlation was found between IRRIV and RFR (r = 0.81). The IRRIV test showed 100% sensitivity and 84% specificity in predicting RFR, with an area under the curve (AUC) of 0.80 in ROC curve analysis. Post-surgery, 3.2% of patients developed AKI and 38.7% developed subclinical AKI. RFR was a predictor of subclinical AKI, with 61% sensitivity and 88.8% specificity, while IRRIV had a 46.1% sensitivity and 100% specificity in predicting subclinical AKI, suggesting that IRRIV is a significant predictor of RFR in patients undergoing cardiac surgery, and both RFR and IRRIV can predict the occurrence of subclinical AKI postoperatively.

Nalesso et al. [14] focused on assessing the RFR in patients with β-thalassemia major (β-TM) using an ultrasound and Doppler method based on IRRIV [11]. Nineteen β-TM patients were enrolled for this pilot study where a strong negative correlation was found between the mean ferritin values and Delta RRI, suggesting that the Renal Stress Test (RST) based on IRRIV is a reliable tool for assessing the presence of RFR in this population. The concept of RFR and its assessment through IRRIV have been the focus of these significant studies that aimed to delve into the potential of IRRIV as a non-invasive reliable method to measure RFR in diverse clinical scenarios, including healthy volunteers, patients undergoing cardiac surgery, and those affected by β-TM. Nalesso et al. [14] named RST the assessment of RFR using the ultrasound technique where IRRIV and Delta IR (RRI during stress—RRI in rest condition—were considered separately as two parameters describing the magnitude of RFR (IRRIV) and its presence (Delta RRI); IRRIV is defined as the percentage difference between baseline RRI and stress RRI while Delta RRI is considered a predictor of RFR if > 0.05. Nalesso et al. have also introduced the IRRIVsc parameter, which accounts for the normalization of the IRRIV value of the patient’s body surface area (BSA) by providing an additional parameter useful for the dynamic assessment of the RFR in subjects who may have significant body weight variations. In conclusion, these studies highlight the versatility and reliability of IRRIV in assessing renal health across varied patient demographics. Its ability to predict AKI and its role in the early detection of subclinical renal impairment are particularly noteworthy. These findings advocate for the broader adoption of IRRIV in routine clinical practice for early renal health assessment and management.

The aim of this pilot study was to evaluate the sample size, feasibility, and methodological issues of a future study about the possible role of IRRIV-based RST [14] as a marker of RFR in a group of living kidney transplant donors. Finally, we would like to fortify the rationale of a future study, evaluating its consistency in a small cohort of donors by its correlation with creatinine levels and estimated Glomerular Filtration Rate (eGFR) pre- and 12 months post-donation.

## 2. Materials and Methods

This observational, monocentric, and unfunded study conducted at the Padova University Hospital (Kidney and Pancreas Transplant Unit and Nephrology, Dialysis and Transplant Unit), included 11 preliminary patients who were scheduled for and underwent minimally invasive laparoscopic surgery to donate their kidneys for living donor kidney transplantation. This study was conducted in accordance with the World Medical Association’s Helsinki Declaration ethical principles. Ethical review and approval were waived for this study as required due to the clinical investigation; the local ethics committee was informed (protocol number AOP3141 at Comitato Etico Territoriale Area Centro-EST Veneto, Italy). The data anonymization process prevented any possible transmission of sensitive data, saving subject privacy. Each subject underwent a comprehensive evaluation for kidney donation eligibility, adhering to the National Transplant Center’s standards and the 2017 KDIGO guidelines. This evaluation included an extensive medical history review, a series of blood and urine tests, infectious disease screenings, diagnostic and specialized examinations, and gender-specific assessments, along with the necessary legal and psychological evaluations according to Italian laws. Exclusion criteria were standard criteria and or eGFR below 60 mL/min/1.73 m^2^, renal diseases or systemic syndromes affecting the kidneys. Additionally, any condition contraindicating a temporary increase in intra-abdominal pressure, like recent abdominal surgery, was also a criterion for exclusion from this study as all patients underwent an in-depth ultrasound study including RST in which the IRRIVs were assessed the day before surgery and 12 months later, according to the local clinical practice. The study of IRRIVs was conducted according to a previously published method [11] and called RST [14] for easier clinical and experimental understanding and interpretation. This examination was performed with Esaote MyLab Gamma with a AC2541 convex probe (Esaote, Genoa, Italy) and always by the same expert operator, certified for ultrasound by specific training in Color and Doppler techniques, on the same kidney pre- and post- nephrectomy for donation. The ultrasound operator was trained in Ultrasound and ColorDoppler during his master’s degree, and the same ultrasound machines were used in manual mode for measuring RRIs. All these expedients were introduced to minimize the possibility of error in the RRI measurements. RST is included as a routine examination of kidney donors for the purpose of analyzing RFR as a local best clinical practice regardless of research purposes.

### 2.1. Statistical Analysis

All continuous variables were reported as a median and interquartile range (IQR); categorical variables were described by percentages. All variables were evaluated using the Shapiro–Wilk test to assess the normal distribution. A paired *t*-test and Wilcoxon Signed-Rank test were used to compare the level of the markers before and after kidney nephrectomy, as appropriate. All reported *p*-values were two-sided, and statistical significance was set at *p* < 0.05. Statistical analysis was performed with SPSS version 29.0.1.0 (SPSS Inc., Chicago, IL, USA).

No previous studies about the possible role of IRRIV-based RST in living kidney transplant donors have been reported. We adopted the rule of thumb of [15], who suggested between 10 and 40 per arm.

We performed an estimation of the sample size for a future study using the OpenEpi calculator (https://www.openepi.com) for comparing two means with a power of 80%, according the following formula:

n = [(σ12 + σ22) (Z1-α/2 − Z1-β)2]/Δ2

The notation for the formula: n = sample size, σ1 = standard deviation before kidney donation, σ2 = standard deviation after kidney donation, Δ = difference in means before and after donation, Z1-α/2 = two-sided Z value (e.g., Z = 1.96 for 95% confidence interval). Z1-β = power.

### 2.2. Feasibility Criteria

We considered the current study design adequate in terms of feasibility when at least 90% of enrolled patients completed all procedures, performing the preliminary and subsequent RST. Furthermore, as pilot study, we aimed to identify potential study design issues with the adequacy of instrumentation and data collection, according to [16].

## 3. Results

Demographic characteristics and kidney function (creatinine and eGFR-EPI) at the kidney donation are shown in Table 1.

We analyzed 11 patients before and after the kidney donation, of which 3 (27.3%) were male. One patient suffered from hypertension. In our cohort, we observed a significant eGFR reduction after 12 months from kidney donation and a corresponding increase of creatinine with *p* = 0.003 and <0.001, respectively. Furthermore, we detected a contextual significant reduction of Delta IR (*p* = 0.017). All test results are reported in Table 2.

## 4. Discussion

RFR represents the capacity of the kidneys to increase GFR in response to physiological or pathological stimuli [2,3]. This concept is particularly relevant in the context of living kidney donation, as it provides insights into the donor’s renal compensatory ability post nephrectomy. Evaluating RFR in potential living kidney donors is crucial as a high RFR indicates a robust renal compensatory response, which is a favorable prognostic factor for the donor’s long-term renal function. Conversely, a low RFR may signal a limited renal reserve, posing a higher risk of developing CKD post donation [17]. Clinical studies have shown that most living kidney donors maintain normal renal function over the long term, but a subset develops CKD. Factors like pre-donation RFR can help identify individuals at higher risk [18] of CKD or AKI post-surgery.

RFR is typically measured by calculating the difference between baseline GFR and peak GFR following a protein load or pharmacological stimulation (e.g., dopamine [19] or amino acid infusion) with not always useful and unambiguous results for the screening of patients, especially in the living donor cohort. The most commonly used method for GFR assessment is the clearance of filtration markers such as inulin, iothalamate, or iohexol. RFR could provide additional information on kidney health and renal function prognosis. Despite longstanding interest in the RFR as a biomarker in nephrology, its underlying mechanisms remain inadequately understood. Moreover, no consensus has been reached on how it should be quantified. Previous studies on RFR have used various measurement methods and yielded heterogeneous results [20]. The mechanisms underlying RFR are not completely clear although some hypotheses have been proposed and validated by previous studies. One theory is the recruitment of “dormant cortical nephrons”, which are not active during rest but may engage during stress. Another proposed mechanism is glomerular hyperfiltration. Studies have shown that the filtration fraction often remains constant, suggesting changes in blood flow as a primary mechanism for GFR increase [21,22]. However, some researchers have identified an increase in the transcapillary hydraulic pressure gradient as a contributing factor [23,24]. Despite these varying theories, a common underlying mechanism appears to be afferent vasodilation [22,23,24]. This is supported by the observed temporal changes in renal hemodynamics post-protein load and the subsequent peak in the GFR. The increase in the GFR typically occurs around 2 to 2.5 h after the protein load, following changes in renal hemodynamics within the first hour.

Recently, more practical methods like creatinine clearance or estimation equations have been utilized, though they might offer less precision. It is widely accepted that animal protein ingestion, which acts as a stress test for kidneys, can cause a significant increase in the GFR primarily through changes in renal hemodynamics [5,6] resulting in the afferent arteriole vasodilation. This increase in GFR in response to such stress tests is what defines the RFR. Research has shown that RFR, although still present, diminishes progressively in more advanced stages CKD [19,22,23,25] as well as in older individuals. However, in certain conditions of hyperfiltration, such as diabetic nephropathy, GFR might already be at its maximum and therefore does not further increase in response to a protein load. Hyperfiltration conditions, therefore, result in an almost complete utilization of the RFR by not allowing the kidney to be able to recruit it during states of stress exposing patients to stress damage, and which, depending on its magnitude, can result in the onset of AKI or the evolution of kidney damage into CKD.

The IRRIV test involves a mechanical abdominal stress applied externally to compress renal arteries and veins, thereby reducing blood flow [11]. This action activates the kidney’s autoregulation mechanism, which in turn causes afferent vasodilation. The purpose of this vasodilation is to maintain glomerular perfusion despite the reduced blood flow caused by the external pressure [26]. The afferent vasodilation is a key response in the mechanical stress test (IRRIV) [11,12]. This vasodilation can be assessed using color Doppler (CD) ultrasound in the case of the IRRIV test. The RRI is a widely used method for evaluating blood flow in kidney vessels to calculate the relationship between systole and diastole, serving as an indicator of flow resistance within the kidney [27]. Therefore, RRI is a valuable tool for assessing changes in renal perfusion; specifically, in this context, a decrease in RRI in an interlobular artery in a patient can be interpreted as an indirect measurement of pre-glomerular vasodilation [11]. Thus, as previously demonstrated, IRRIV can be used to study RFR [11,12,13,14] by providing useful information on the possible development of AKI after surgery and the possible evolution of AKI into CKD after kidney donation.

The preliminary results obtained from our pilot study (Table 2) show that RST can be performed 12 months after kidney donation and the levels of creatinine and eGFR CKD-EPI vary. The average creatinine rises from a mean value of 70.3 to 98.3 µmol/L, with a parallel median reduction in eGFR from 104 to 66 mL/min/1.73 m^2^. These results can be explained by a reduction in the RFR due to the halving of the nephron mass following nephrectomy. In fact, the subject at 12 months has a nephron mass equal to 50% of the previous pre-donation kidney mass. Interestingly, the demonstration that the RST shows a reduction in Delta RRI, due to the reduction in the capacity of vasodilation of the afferent arteriole under stimulus, may indicate a loss of RFR when compared to the pre-donation condition. Part of the potential vasodilation is lost due to the basal utilization of the RFR for maintaining metabolic balance in the patient as the recruitment of the RFR in basal conditions occurs with less afferent vasodilation during the RST. The reduction in nephron mass induces adaptive mechanisms in the remaining kidney that result in both the recruitment of RFR and hyperfiltration of the remaining nephrons. Both of these alterations determine greater efferent arterial vasodilation in basal conditions compared to the pre-donation situation. This hemodynamic situation is reflected in a reduction in Delta RRI at 12 months.

This correlation between the Delta RRI and RFR may represent important implications in the diagnosis of subclinical kidney dysfunction or underlying CKD [25]. In fact, while CKD stages 3, 4, and 5 can be easily diagnosed by a reduction in GFR, stages 1 and 2 often present a normal baseline GFR and the diagnosis may only be made using a stress test such as protein loading. The possibility of investigating the RFR before and after donation with RST makes it possible to monitor the adaptive hemodynamic changes of the kidney to nephron mass loss over time, giving important information on the possible development of AKI in the immediate post-operative period or long-term CKD.

The purpose of this pilot study was to verify the possibility of using the RST in the population of kidney donor subjects. We achieved the feasibility criteria and now we can project a future study with an adequate number of cases equal to 48. In all patients analyzed with the RST, no clinical complications or technical problems with the execution of the ultrasound examination were identified. In particular, the placement of a weight equal to 10% of the patient’s body weight on the anterior abdominal wall [11] did not cause discomfort or complications in all of the patients analyzed for the IRRIV test. The RST in the living donor population was found to be easy, safe, and reproducible over time.

Furthermore, considering pathophysiology issues, kidney donors can be considered healthy given their eligibility for donation. Being healthy subjects, according to previous literature reports [11,12], they can be studied with the IRRIV test to analyze their RFRs. Once the donation is made, these patients present a halving of the nephron mass with physiological adaptive hemodynamic responses of the surviving kidney, which must recruit the RFR and induce hyperfiltration of the residual nephrons to maintain metabolic compensation.

This study has limitations: First, the major limitation of our study lies in the number of cases analyzed, which reduces its statistical validity for the possible identification of significant correlations also for the IRRIV and the IRRIVsc. Our results refer to a selected donors population and thus may not be fully generalizable. They should be considered hypothesis-generating, and need to be confirmed in further studies. Second, ultrasound quality is highly dependent on the ultrasound practitioner. In our study however, to minimize these subjective limitations, especially during measurements, only one Nephrologist performed the examinations with the same ultrasound machine using the manual measurements to calculate the RRIs. Third, RST perfomed by the application of a weight equal to 10% of the patient’s body weight on the donor abdomen may be not tolerated. However, in our small sample, all donors tolerated the test. This study also has strengths. The sssessment of RST with IRRIV can be easily applied at the donor’s bedside in a non-invasive and reproducible way.

The preliminary data of this study demonstrate the possibility of applying the RST in the living donor population, opening up its use in common clinical practice to evaluate eligibility for donation even in patients in whom a borderline renal situation is identified with eGFR CKD-EPI or doubtful creatinine levels.

In nephrology, renal function assessment and staging of AKI and CKD predominantly rely on serum creatinine (sCr) values and sCr-based equations. The CKD-EPI equation, a more recent advancement, addresses some limitations of the older MDRD equation but still has its challenges [28]. Particularly, it underestimates GFR in diabetic patients [29] and overestimates it in the elderly compared to other methods like the Cockcroft–Gault formula [30] or 24-h measured creatinine clearance (CrCl) [31]. Recognizing these limitations, physicians have started incorporating other clinical predictors, including AKI biomarkers [32], especially in critical care settings. These biomarkers aid in the timely diagnosis of renal damages, allowing for early intervention to prevent further impairment. However, they cannot predict kidney damage before exposure to stress, as in the case of kidney donation. Currently, kidney stress tests, which are crucial in such evaluations, are cumbersome and limited to certain centers, like transplant centers performing protein loading tests on living kidney donors. The RST test presents a promising alternative in this regard as the necessity of evaluating the RFR in living donors stems from the growing reliance on living kidney transplantation as a definitive therapy for end-stage renal disease. With the increasing demand for donor organs and the inherent risks associated with living donation, thorough preoperative assessment, including RFR, becomes crucial for ensuring donor safety and optimal recipient outcomes. Current guidelines by organizations such as the American Society of Nephrology and the European Renal Association recommend assessing RFR as part of the comprehensive evaluation of living kidney donors.

## 5. Conclusions

Assessing the Renal Functional Reserve (RFR) in living kidney donors seems to be a promising tool for predicting post-donation renal outcomes. In our preliminary observational study, we demonstrated the feasibility of the use of the Intra-Parenchymal Renal Resistive Index Variation (IRRIV) test to measure RFR in living donors. Moreover, we found that the RST using IRRIV in the living donor population can provide information about kidney function pre-donation when the markers do not detect the exact loss of renal function reserve. This study suggests the future role of a Renal Stress Test (RST) in assessing the RFR in living kidney donors. A RST could provide a comprehensive understanding of a donor’s renal health and compensatory mechanisms post-nephrectomy and identify donors at a higher risk of adverse renal consequences. Such assessments are instrumental in facilitating informed decision-making and personalized risk assessment for potential donors in a non-invasive and reproducible way.

This preliminary study presents some limitations due to the small sample size of only 11 patients, which reduces the statistical power and generalizability of findings. Additionally, the single center limits external validity. The lack of a control group for comparison limits the ability to make definitive conclusions. Future randomized controlled trials would strengthen the evidence. Finally, the short follow-up period of 12 months post-nephrectomy may not capture long-term outcomes. 

This pilot study paves the way for further research to validate and refine this approach and can be considered a foundational step toward integrating RFR assessment in routine clinical practice for living kidney donors.

## Figures and Tables

**Table 1:** Demographic and clinical renal data at the kidney donation.

	Median (IQR)
Age (years)	52 (46–56)
Height (cm)	165 (160–170)
Weight (kg)	62 (56–88)
Pre-operative creatinine (mmol/L)	70.3 ± 17.2
Pre-operative eGFR (mL/min/1.73 m^2^)	104 (78–107)

**Table 2:** Statistical analysis of serum creatine, eGFR-EPI, IRRIV, and Delta IR before and after kidney donation.

	Before Nephrectomy	12 Months after Nephrectomy	*p*
Creatinine(µmol/L)	70.3 ± 17.2	98.3 ± 13.8	<0.001 ^
eGFR CKD-EPI (mL/min)	104 (78–107)	66 (60–81)	0.003 °
Mean IR	0.61 (0.54–0.63)	0.57 (0.54–0.59)	0.27 °
IRRIV %	14.29 (11.28–18.15)	12 (7.3–15.25)	0.57 °
IRRIV Sc %	8 ± 4.2	7.3 ± 4.4	0.993 ^
Delta IR	0.083 (0.075–0.148)	0.073 (0.041–0.097)	0.017 °

^ Paired *t*-test for normally distributed variables; ° Wilcoxon Signed-Rank test for no normally distributed variables; IRRIV Sc: IRRIV normalized for corporeal surface; On the base of previous results, we esteemed the sample size of the future project, we need a sample size of 48 donors.

## Data Availability

Data is contained within the article.
